# Simultaneous Enhancement of Strength and Sulfide Stress Cracking Resistance of Hot-Rolled Pressure Vessel Steel Q345 via a Quenching and Tempering Treatment

**DOI:** 10.3390/ma17071636

**Published:** 2024-04-03

**Authors:** Jing Zhang, Ming-Chun Zhao, Yan Tian, Jimou Zhang, Zhen Wang, Ying-Chao Zhao, Longsheng Peng

**Affiliations:** 1 College of Automotive Engineering, Hunan Industry Polytechnic, Changsha 410082, China; 2 School of Materials Science and Engineering, Central South University, Changsha 410083, China; 3 Xiangtan Iron & Steel Co., Ltd. of Hunan Valin, Xiangtan 411101, China; 4 Hunan Lifang Roll Co., Ltd., Hengyang 421681, China

**Keywords:** pressure vessel steel, Q345, strength, sulfide stress cracking (SSC), quenching and tempering (Q&T) processing

## Abstract

Sulfide stress cracking (SSC) failure is a main concern for the pressure vessel steel Q345 used in harsh sour oil and gas environments containing hydrogen sulfide (H_2_S). Methods used to improve the strength of steel usually decrease their SSC resistance. In this work, a quenching and tempering (Q&T) processing method is proposed to provide higher strength combined with better SSC resistance for hot-rolled Q345 pressure vessel steel. Compared to the initial hot-rolled plates having a yield strength (YS) of ~372 MPa, the Q&T counterparts had a YS of ~463 MPa, achieving a remarkable improvement in the strength level. Meanwhile, there was a resulting SSC failure in the initial hot-rolled plates, which was not present in the Q&T counterparts. The SSC failure was not only determined by the strength. The carbon-rich zone, residual stress, and sensitive hardness in the banded structure largely determined the susceptibility to SSC failure. The mechanism of the property amelioration might be ascribed to microstructural modification by the Q&T processing. This work provides an approach to develop improved strength grades of SSC-resistant pressure vessel steels.

## 1. Introduction

Currently, the increasing demand for oil and natural gas promotes active attention to pressure vessels which are important equipment for the separation, storage, and transportation of oil and natural gas in the energy industry [1,2,3,4]. Steel is one of the most commonly used materials for the production of pressure vessels [5,6,7]. Among those steels used for pressure vessels, Q345 steel, available in China, is a typical low carbon and low alloy hot-rolled steel with moderate yield and tensile strength that is extensively used in low- and medium-pressure vessels owing to its low cost (free of the precious alloy elements) and decent tensile strength (ultimate tensile strength (UTS) of ~470 MPa, yield strength (YS) of ~345 MPa) [8,9,10,11]. In the commercial use of the pressure vessel steel Q345 in harsh sour oil and gas environments containing hydrogen sulfide (H_2_S), the sulfide stress cracking (SSC) failure that is caused by the combined action of both the H_2_S and tension stress has gained considerable attention due to the possible resulting disastrous consequences. There has been no other such phenomenon as SSC both resulting in enormous financial loss and attracting much attention in the field of pressure vessel steels. Paying attention to SSC failure is very valid for this steel when it is used in sour oil and gas environments containing H_2_S. The direct industrial importance of SSC resistance in Q345 pressure vessel steel is best emphasized by its extensive use for the exploration and exploitation of oil and natural gas in the energy industry.

Driven by cost considerations, higher pressure and larger diameter pressure vessels are favored in the oil and gas industries, which are usually realized by increasing the strength of the pressure vessel steels. There is, thus, a resulting strong desire to improve the strength and SSC resistance of the pressure vessel steel Q345 as used in harsh, wet H_2_S environments. However, the strength of the steel significantly determines its SSC susceptibility, and the strength increases usually simultaneously with decreasing the SSC resistance for the steel [12,13,14,15]. Increasing the strength will put higher requirements on the SSC resistance, and it has long been difficult to strike a good balance between strength and SSC resistance in steels. Approaches aimed at developing improved strength grades of pressure vessel steels must take into account the issue of their SSC susceptibility. Thus far, there have been numerous attempts to develop improved strength grades of pressure vessel steels with good SSC resistance in sour oil and gas environments containing H_2_S [16,17]. These attempts were usually performed by designing new chemical compositions and/or by improving the hot-rolled processing, which might require the renewal of the rolling equipment, which is a little economically unfeasible for industrial manufacturers.

In this work, an additional quenching and tempering (Q&T) heat treatment was introduced to the hot-rolled pressure vessel steel Q345 for the simultaneous improvement of both the strength and the SSC resistance, without a requirement for change to the chemical composition or to the hot-rolled processing and the rolling equipment. The mechanism by which the Q&T processing influenced the strength and the SSC resistance was analyzed. The mechanism of the property amelioration might be ascribed to microstructural modification by the Q&T processing, i.e., the elimination of the banded structure. In pressure vessel technology, the actual industrial manufacturing procedure has been mainly established on the production experience, and few attempts have been made with this enhanced Q&T approach to develop SSC-resistant pressure vessel steels with a high strength level. This should also in turn help to improve the manufacture of pressure vessel steels with improved strength grades that are resistant to SSC failure.

## 2. Experimental Procedures

The experimental materials were 10 mm thick hot-rolled plates of typical Q345 pressure vessel steel with a chemical composition (wt.%) of 0.17 C, 0.29 Si, 1.24 Mn, 0.008 P, 0.001 S, 0.03 (Nb + V + Ti), 0.1 Cu, 0.03 Cr, 0.02 Ni, and 0.042 Al, which were provided by Xiangtan Iron & Steel Co., Ltd. of Hunan Valin, China. The rolling schedule was set as a two-stage (recrystallized austenite region + non-recrystallized austenite region) controlled rolling process [18]. Hot-rolled plates were cooled immediately at ~5 °C/s to room temperature (RT) after rolling. All the experimental samples (including the initial hot-rolled samples and the Q&T samples) were wire-cut from the center part of the hot-rolled plates. The Q&T samples were water-quenched after austenitizing at 890 °C for 1 h and tempered at 700 °C for 3 h followed by water-cooling.

The microstructures in the transverse sections were characterized using a scanning electron microscope (SEM) with energy-dispersive X-ray spectroscopy (EDS). Specimens for SEM observation were usually ground, polished, and etched in a nital solution of 2 pct. Specimens for electron backscatter diffraction (EBSD) observation were usually ground, polished, and then electrochemically polished by a solution (90 vol% acetic acid + 10 vol% perchloric acid) at 40 V. The EBSD data were detected by a field emission SEM (LEO-1550 Schottky, Carl–Zeiss, Jena, Germany) equipped with an orientation imaging microscope (OIM) system from TexSEM Laboratories, Inc., Provo, UT, USA, with a ~30 nm spot size at 25 kV. The EBSD scan step was 0.1 µm. A tensile test was performed at RT temperature with a 5 mm/min cross-head speed and was conducted three times to obtain an average value for every material condition. The hardness of the different microstructural components in the microstructures containing the banded structure was tested by a microhardness tester using a 2 N load and a 10 S load time. The hardness indents were conducted ten times to obtain an average value. An SSC test was performed using the uniaxial constant load test with CORTEST (0–200,000 psi) proof-ring devices according to the NACE TM 0177-2005: Method A—uniaxial constant load test [19]. The specimens had a gauge length of 25.4 mm and a gauge diameter of 6.35 mm, which were carefully machined and ground in the gauge section to avoid overheating and cold working. During the machining, the last two passes removed less than 0.05 mm. The surface roughness was ~0.25 µm after mechanical polishing. The specimens were degreased and rinsed using acetone before testing. The gauge section was not contaminated after cleaning. The specimens, with an imposed uniaxial tensile stress of 85% actual yield strength value, were immersed in Solution A, which was saturated by 0.1 MPa H_2_S at RT temperature. The test was terminated after 720 h or at failure occurrence, whichever occurred first. The specimen was thought to pass if there was no failure until 720 h. The evaluation of the SSC resistance was conducted using three specimens for every material condition. If any of those three specimens failed to pass, the corresponding material condition did not pass. After the SSC test, the morphologies of the specimens were observed by an optical microscope (OM), SEM, and digital camera.

## 3. Results

### 3.1. Microstructures

**Figure 1:**
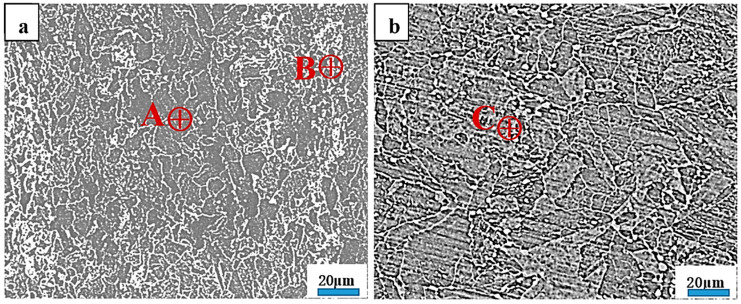
SEM microstructure characteristics: (**a**) initial hot-rolled plates; (**b**) Q&T counterparts.

Figure 1 shows the SEM microstructures of the initial hot-rolled plates and the Q&T counterparts, revealing a pronounced difference in the microstructure characteristics. The SEM microstructure in the initial hot-rolled plates, as shown in Figure 1a, contained a ferrite matrix plus visible banded structures. The region of the ferrite matrix is gray, and the region of the banded structure is bright white. Many carbides were agglomerated randomly in the banded structures. Meanwhile, the SEM microstructure in the Q&T counterparts, as shown in Figure 1b, was more uniform and contained a ferrite matrix plus numerous dispersed carbides, showing full elimination of the banded structures, which indicated a typical tempered martensite defined in the carbon steels subjected to Q&T processing [20]. At least five points were analyzed in the EDS results for every similar region. Although the compositions could not be precisely detected by the EDS measurement, the obtained results are semi-quantitative data and can roughly reflect the composition distribution characteristics in the microstructures. Point A and Point B in Figure 1a and Point C in Figure 1b are highlighted as representatives. Their corresponding compositions evaluated using the EDS spectra are listed in Table 1. Point A contained ~1.2 wt.% C, Point B contained ~5.8 wt.% C, and Point C contained ~1.5 wt.% C.

**Figure 2:**
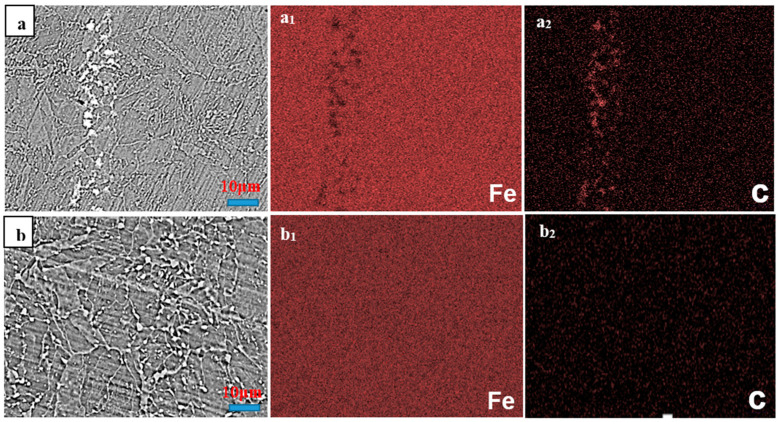
SEM micrograph (**a**) and X-ray maps for (**a_1_**) Fe and (**a_2_**) C in the initial hot-rolled plates; SEM micrograph (**b**) and corresponding X-ray maps for (**b_1_**) Fe and (**b_2_**) C in the Q&T counterparts.

Figure 2 shows the SEM-EDS results for the initial hot-rolled plates and the Q&T counterparts. The X-ray maps for the C and Fe of Figure 2a, in the initial hot-rolled plates, are exhibited in Figure 2a_1_,a_2_, and those of Figure 2b, in the Q&T counterparts, are exhibited in Figure 2b_1_,b_2_. The carbon was distributed largely along the banded structures in the initial hot-rolled plates, while being relatively evenly distributed throughout the ferrite matrix in the Q&T counterparts. Therefore, the carbon had an enriched distribution in the banded structures of the initial hot-rolled plates, while showing a relatively uniform distribution in the Q&T counterparts.

This carbon-enriched banded structure in the initial hot-rolled plates was largely attributed to the segregation of the component in the hot-rolled plates. It is well known that the carbon element in steels easily produces segregation in the microstructure. After the hot rolling, the segregated carbon element was distributed into the strips, presenting an alternate carbon-poor plus carbon-rich austenite distribution. The ferrite appeared in the carbon-poor austenite bands preferentially because of its higher critical temperature (Ar_1_ temperature), which would further repel the carbon element into the neighboring carbon-rich austenite bands. Eventually, the residual carbon-rich austenite band transformed during the following continuous cooling, and the transformed microstructure was carbon-rich. Consequently, phase transition during the hot rolling was closely accompanied by composition partitioning, which directly influenced the distribution and proportion of every phase.

### 3.2. Tensile Properties and SSC Evaluation

**Figure 3:**
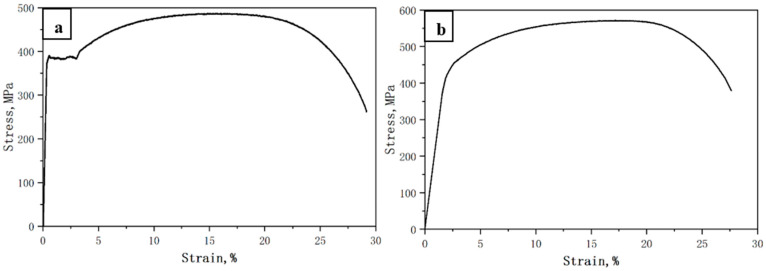
Representative engineering stress–strain curve at RT temperature: (**a**) initial hot-rolled plates; (**b**) Q&T counterparts.

Figure 3 depicts the representative engineering stress–strain curve tested at RT temperature for the initial hot-rolled plates and the Q&T counterparts. The initial hot-rolled plates exhibited an evident yield point. The Q&T counterparts exhibited a continuous yielding behavior. Table 2 lists the tensile properties at RT temperature for the initial hot-rolled plates and the Q&T counterparts, which includes the yield strength (YS), the ultimate tensile strength (UTS), and the elongation (EL). The initial hot-rolled plates had a YS of ~372 MPa, a UTS of ~486 MPa, and an EL of ~30%. In contrast, the Q&T counterparts had a YS of ~463 MPa, a UTS of ~569 MPa, and an EL of ~28%, reflecting an ~25% YS increase. Compared to the initial hot-rolled plates, those that underwent Q&T processing achieved a remarkable improvement in the strength level. This strength level improvement can be attributed to the tempered martensite microstructure. As generally documented [21,22,23], a tempered temperature has significant effects on quenched martensite. In the present work, tempering at 700 °C for the Q&T processing largely promoted the desolvation of supersaturated carbon atoms from the quenched martensite matrix, contributing to carbide formation in the tempered martensite and thus leading to the resulting precipitation strengthening. As for the insignificant loss in elongation of the Q&T counterparts, it can be attributed to the relatively homogenized Q&T microstructure with fully eliminated banded structures.

Table 3 lists the SSC results for the initial hot-rolled plates and the Q&T counterparts. For the initial hot-rolled plates, two of the three specimens cracked; thus, this specimen group did not pass. As for the Q&T counterparts, the three specimens did not crack in the fixed time, i.e., 720 h, and hence this specimen group passed the SSC test. As there was SSC failure in the initial hot-rolled plates but not in the Q&T counterparts, it can be concluded that the SSC resistance of the initial hot-rolled plates was lower than that of the Q&T counterparts, despite the fact that the latter exhibited a higher strength.

## 4. Discussion

**Figure 4:**
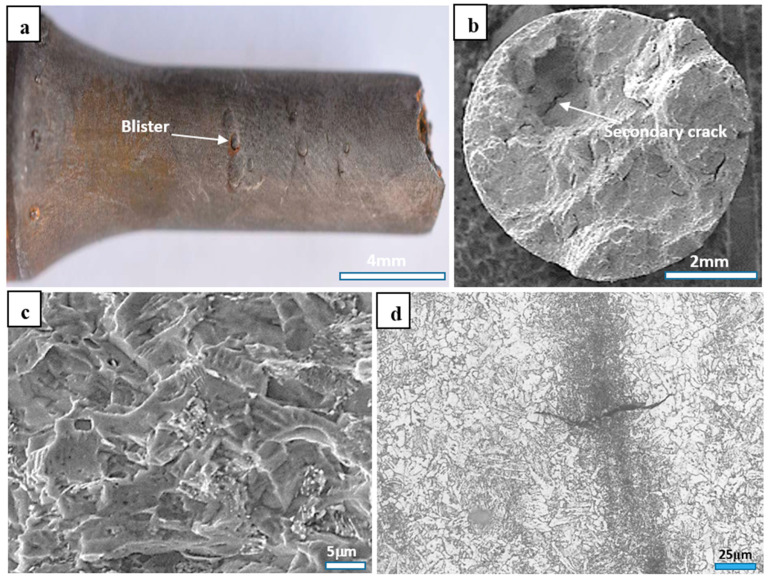
(**a**) Hydrogen blister on the surface; (**b**) secondary crack and (**c**) predominant quasi-cleavage on the fracture; (**d**) crack perpendicular to the carbon-rich band.

Usually, steel that has a higher strength or hardness presents worse SSC resistance [12,13,14,15]. However, for this experimental steel, the Q&T counterparts gave a higher YS and UTS while showing better SSC resistance. Hence, the SSC failure was not only determined by the strength for the present experimental steel. The usual viewpoint on the SSC of steel is that hydrogen penetration can happen under the condition of the release of hydrogen. It is well known that the SSC mechanism is attributed to the susceptibility of the hydrogen embrittlement (HE) [24,25,26,27,28,29], which could also be substantiated by the evidence revealed in the present work shown in Figure 4, where Figure 4a shows blisters of hydrogen on the outside surface in the cracked specimen, Figure 4b shows secondary cracks on the fracture surface in a cracked specimen, and Figure 4c shows the predominant quasi-cleavage characteristic on the fracture surface in a cracked specimen. Therefore, the SSC failure of the present experimental steel agreed with the hypothesis on the embrittling contribution by hydrogen, i.e., decided by the hydrogen permeation-controlled mechanism [24,25,26,27,28,29]. The SSC mechanism involved chemical reactions under the co-action of H_2_S corrosion and tensile stress as follows. During the SSC test, the atomic hydrogen was caused by:

$$H_{2}S+Fe\to FeS+2H$$

(anodic reaction: $Fe-2e\to {Fe}^{2+}$; cathodic reaction: $2H^{+}+2e\to 2H$).

As hydrogen atoms were cathodically produced on the steel surface, the presence of H_2_S promoted their penetration into the specimen. The penetrated hydrogen atoms tended to diffuse into sites with segregations, inclusions, coarsening carbides, and banded structures, i.e., so-called hydrogen traps. The effective hydrogen concentration (C_H-Effective_) was involved in the hydrogen traps in the microstructure of the specimens, which were prone to adsorbing and trapping the penetrated hydrogen. SSC failure occurred as a critical hydrogen concentration (C_H-Critical_) was reached [30].

The Q&T processing produced an evidently different microstructure from the initial hot-rolled plates—that is, enriched carbon was present in the banded structures in the initial hot-rolled microstructure, while it showed a relatively uniform distribution in the Q&T microstructure with full elimination of the banded structures. The SSC results indicated that SSC failure only occurred in the initial hot-rolled SSC test specimens, while it did not occur in the Q&T SSC test specimens. Therefore, the SSC failure was inevitably related to the microstructures. SSC failure of steels related to hydrogen penetration is identified as often happening at sites of hydrogen congregation. Finally, the heterogeneous micro-zones in the microstructure, i.e., the carbon-enriched banded structures, in the initial hot-rolled microstructure became the main trap sites for hydrogen, resulting in the exacerbation of the crack at the critical value. The SSC failure mechanism of the hot-rolled steel was largely related to the homogeneity in the structure, i.e., the carbon-enriched banded structures. As is well known, the coefficient of hydrogen diffusion decreases with increasing the carbon content in the microstructures of steels [31,32]. The banded structure in the initial hot-rolled microstructure was carbon-rich, as stated above. Therefore, the coefficient of hydrogen diffusion of the carbon-rich banded structure would be lower than that of the ferrite matrix. When the penetrated atomic hydrogen spread into the carbon-rich banded structure from the ferrite matrix, the coefficient of hydrogen diffusion changed from high to low. Therefore, the hydrogen accumulated in the carbon-rich banded structure, which trapped and aggregated the hydrogen atoms, and the C_H_ reached the C_H-Critical_ preferentially; accordingly, SSC failure occurred. The relationship between the crack and the carbon-rich banded structure is substantiated in Figure 4d, where the crack is perpendicular to the carbon-rich banded structure, with a relatively wider gap in the middle of the crack that is directly located in the carbon-rich banded structure.

In comparison with the initial hot-rolled microstructure, the banded structure susceptible to SSC failure was fully eliminated in the Q&T microstructure, and so the detrimental influence on the SSC resistance from the aggregated cementites was almost negligible. The improvement in the uniformity in the microstructure helped to ameliorate the SSC resistance. As mentioned, numerous fine carbides were dispersed in the whole ferrite matrix in the Q&T microstructure. Although certain kinds of hydrogen traps such as fragmentized or aggregated layered cementites, i.e., carbon-rich bands, might act as initiation sites of SSC failure, it was proposed that the harmful influence of hydrogen might be ameliorated by the valid distribution of benign traps. Such cases would either inhibit the accumulation of hydrogen at the sites of the incipient failure by holding a relative homogeneous distributed hydrogen profile or delay the SSC onset to a higher concentration for a longer time. Based on previous studies [12], dispersed fine precipitates acting as unharmful traps of hydrogen in steels might provide a large number of sites for the redistribution of hydrogen and thus might change the critical condition of SSC occurrence. Keeping in mind such a consequence, banded structure elimination in Q345 pressure vessel steel is beneficial to improve its SSC resistance.

**Figure 5:**
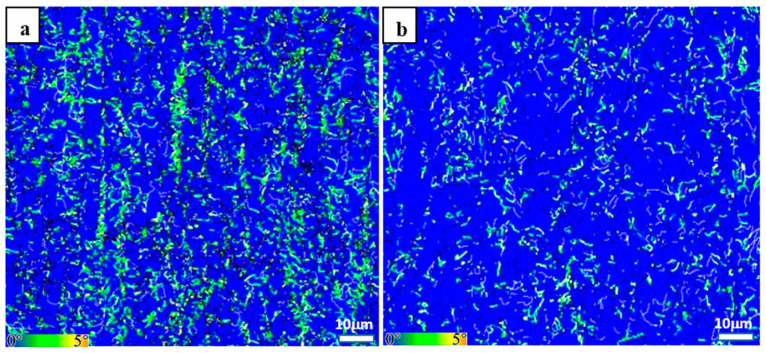
KAM maps: (**a**) initial hot-rolled plates; (**b**) Q&T counterparts.

Another important factor for triggering SSC failure is residual stress [33,34,35], which is closely related to the plastic deformation accompanied by phase transformation. A Kernel average misorientation (KAM) map of the EBSD that reveals local misorientation and the strain energy may be used to estimate the stress distribution [36,37,38]. Figure 5 shows a typical KAM map, in which the green area indicates the strain induced by a high dislocation density. Obvious strain was retained in the initial hot-rolled microstructure with the distribution of a certain band, while there was a sharp reduction in the stress concentration in the Q&T counterparts. As mentioned above, carbides were agglomerated in the banded structures in the initial hot-rolled microstructure, thus inhibiting ferrite growth and resulting in a high distortion energy in these locations [39,40]. Meanwhile, the Q&T processing relieved the residual stress greatly. Therefore, Q&T processing could improve SSC resistance by decreasing residual stress. In addition, it is recognized that SSC failure only occurs in steel with a hardness value higher than 22 HRC or 248 HV; otherwise, the peak C_H_ value at high-concentration regions is insufficient to generate SSC failure [41,42,43]. The measured hardness values of the matrix and the carbon-rich banded structures for the initial hot-rolled specimens corresponded to ~190 HV and ~260 HV, respectively, while that of the matrix for the Q&T specimens corresponded to ~220 HV. From this perspective, the preferential occurrence of SSC failure in the carbon-rich banded structures was a high probability.

## 5. Conclusions

This work introduced Q&T processing to Q345 hot-rolled pressure vessel steel. The following conclusions were obtained:

1. Higher strength combined with better SSC resistance was obtained. Compared to the initial hot-rolled plates having a YS of ~372 MPa, the Q&T counterparts had a YS of ~463 MPa, achieving an increase of ~25%. Furthermore, SSC failure occurred in the initial hot-rolled plates, while no SSC failure occurred in the Q&T counterparts.
2. The SSC failure was not only determined by the strength. Carbon-rich zones, residual stress, and sensitive hardness in the banded structure largely determined the susceptibility to SSC failure. The elimination of banded structures was beneficial to improve the resistance to SSC failure.
3. The simultaneous improvement in strength and SSC resistance via Q&T processing for Q345 hot-rolled pressure vessel steel provides a prospect for developing improved strength grades of SSC-resistant pressure vessel steels.

## Figures and Tables

**Table 1:** EDS compositions (wt.%) of Point A and Point B in Figure 1a and Point C in Figure 1b.

	Fe	Mn	C
A	96.1	2.7	1.2
B	92.1	2.1	5.8
C	95.6	2.9	1.5

**Table 2:** Comparison of tensile properties at room temperature.

	YS, MPa	UTS, MPa	EL, %
Initial hot-rolled plates	372 ± 13	486 ± 15	30 ± 2
Q&T counterparts	463 ± 17	569 ± 11	28 ± 2

**Table 3:** Comparison of SSC results.

Speci. Group	Speci. No.	Pass or Not Pass	Pass or Not Pass
Initial hot-rolled plates	1#	N	N
	2#	N	
	3#	Y	
Q&T counterparts	1#	Y	Y
	2#	Y	
	3#	Y	

Note: Y, pass; N, not pass.

## Data Availability

Data are contained within the article.

## References

[1] Liao K.X., Qin M., He G.X., Chen S.J., Jiang X.H., Zhang S.J. (2023). Improvement of integrity management for pressure vessels based on risk assessment-A natural gas separator case study. J. Loss Prev. Process Ind..

[2] Yersak T.A., Elhamid M.A., Dailly A., Rogers M., Prince J., Cai M. (2019). Dynamics of a type IV conformable pressure vessel for natural gas passenger vehicles. Int. J. Press. Vessel. Pip..

[3] Nelson N.R., Prasad N.S., Sekhar A.S. (2023). Structural integrity and sealing behaviour of bolted flange joint: A state of art review. Int. J. Press. Vessel. Pip..

[4] Yoon S.J., Lee H.J., Yoon K.B., Ma Y.W., Baek U.B. (2018). Hydrogen damage in 34CrMo_4_ pressure vessel steel with high tensile strength. J. Mech. Sci. Technol..

[5] Gupta C. (2023). Peak cyclic stress and ratcheting response of a low alloy steel for reactor pressure vessel applications. Metall. Mater. Trans. A.

[6] Buck Z.N., Connolly M.J., Martin M.L., Lauria D., Killgore J.P., Bradley P.E., Chen Y., An K., Slifka A.J. (2023). Effects of mechanical deformation on dislocation density and phase partitioning in 4130 steel. Mater. Sci. Eng. A.

[7] Chekhonin P., Das A., Bergner F., Altstadt E. (2024). Microstructural characterisation of brittle fracture initiation sites in reactor pressure vessel steels. Nucl. Mater. Energy.

[8] Tian Y., Zhang J., Wang Z., Zhou Y., Zhang M., Li X., Zhao M., Atrens A. (2024). Sulfide stress cracking (SSC) of a commercial Q345 pressure vessel steel in a wet H_2_S environment. Mater. Sci. Technol..

[9] Tian Y., Zhang J., Wang Z., Shi Z.Y., Li X., Zhao M. (2024). Effect of cooling rate on sulfide stress cracking resistance of a Q345 pressure vessel steel subject to hydrogen sulfide. Steel Res. Int..

[10] Dong Q., Yang P., Xu G., Deng J.L. (2016). Mechanisms and modeling of low cycle fatigue crack propagation in a pressure vessel steel Q345. Int. J. Fatigue.

[11] Lu S.J., Wang H., Dai P.Y., Deng D.A. (2019). Effect of creep on prediction accuracy and calculating efficiency of residual stress in post weld heat treatment. Acta Metall. Sin..

[12] Zhao M.C., Yang K. (2005). Strengthening and improvement of sulfide stress cracking resistance in acicular ferrite pipeline steels by nano-sized carbonitrides. Scr. Mater..

[13] Nguyen T.D., Singh C., Lee D.H., Kim Y.S., Lee T., Lee S.Y. (2024). Deciphering hydrogen embrittlement mechanisms in Ti6Al4V alloy: Role of solute hydrogen and hydride phase. Materials.

[14] Li Q.D., Ghadiani H., Jalilvand V., Alam T., Farhat Z., Islam M.A. (2024). Hydrogen impact: A review on diffusibility, embrittlement mechanisms, and characterization. Materials.

[15] Mukhopadhyay A., Urkude D.K., Mukhopadhyay G. (2024). Effect of cold work on hydrogen embrittlement of monel-400. J. Fail. Anal. Prev..

[16] Ghosh G., Rostron P., Garg R., Panday A. (2018). Hydrogen induced cracking of pipeline and pressure vessel steels: A review. Eng. Fract. Mech..

[17] Park J.S., Lee J.W., Hwang J.K., Kim S.J. (2020). Effects of alloying elements (C, Mo) on hydrogen assisted cracking behaviors of A516-65 steels in sour environments. Materials.

[18] Zhao M., Hanamura T., Qiu H., Yang K. (2005). Lath boundary thin-film martensite in acicular ferrite ultralow carbon pipeline steels. Mater. Sci. Eng. A.

[19] Zeng T.Y., Zhang S.Z., Shi X.B., Wang W., Yan W. (2021). Effects of the primary NbC elimination on the SSCC resistance of a HSLA steel for oil country tubular goods. Materials.

[20] Borisova Y.I., Mishnev R.V., Tkachev E.S., Kniaziuk T.V., Gaidar S.M., Kaibyshev R.O. (2023). Structure, phase composition, and mechanical properties of a high strength steel with transition carbide η-Fe_2_C. Phys. Met. Metallogr..

[21] Li S.J., He M.Y., Hu G.J., Tan Y.Y., Wang C.D., Jing B., Ping D.H. (2022). Pearlite formation via martensite. Compos. Part B-Eng..

[22] Samuels L.E. (2014). Tempering of martensite. Metallogr. Microstruct. Anal..

[23] Caron R.N., Krauss G. (1972). The tempering of Fe-C lath martensite. Metall. Trans..

[24] Zheng Y.J., Sun H.L., Yan L.C., Pang X.L., Volinsky A.A., Gao K.W. (2022). Review of metal carbide nanoprecipitate effects on hydrogen embrittlement of high strength martensitic steel. Anti-Corros. Methods Mater..

[25] Zhou G.Y., Wang X.T., Cao G.H., Russell A.M., Luo M., Dong X.M., Zhang Z.H. (2023). Effect of double tempering process on sulfide stress cracking susceptibility in API-5CT-C110 casing steel. Corros. Sci..

[26] Kei S.H.S., van Haaften W.M., Britton T.B., Pedrazzini S. (2023). A review of the factors that can increase the risk of sulfide stress cracking in thermomechanical controlled processed pipeline steels. Adv. Eng. Mater..

[27] Lu C.H., Zhang H., Li D.N., Han L.H., Wang J.J., Li J., Qi Y., Li F.P., Tian Y.D. (2023). An investigation of microstructure characteristic and sulfide stress cracking behavior of 110 ksi Cr-Mo grade casing steel. Processes.

[28] Luo M., Zhou G.Y., Shen H., Wang X.T., Li M.C., Zhang Z.H., Cao G.H. (2022). Effect of tempering temperature on microstructure and sulfide stress cracking of 125 ksi grade casing steel. Materials.

[29] Liu L.L., Case R. (2022). The influence of H_2_S on hydrogen absorption and sulfide stress cracking resistance of high strength low alloy carbon steel C110. J. Nat. Gas Sci. Eng..

[30] Shehata M.F., El-Shamy A.M. (2023). Hydrogen-based failure in oil and gas pipelines a review. Gas Sci. Eng..

[31] Okayasu M., Sato M. (2021). Examination of hydrogen diffusivity in carbon steels using a newly developed hydrogen permeation system. Exp. Mech..

[32] Fukuda K., Tojo A., Matsumoto R. (2020). Evaluating solubility and diffusion coefficient of hydrogen in martensitic steel using computational mechanics. Mater. Trans..

[33] Tian Y., Luo Z.R., Zeng T.Y., Shi X.B., Yan W., Zhao M.C. (2024). Microstructural characterizations and sulfide stress cracking susceptibility of a high-strength oil country tubular goods-purpose steel under different quenching conditions. Metall. Mater. Trans. A.

[34] Toribio J., Lorenzo M., Aguado L. (2022). Innovative design of residual stress and strain distributions for analyzing the hydrogen embrittlement phenomenon in metallic materials. Materials.

[35] An D.Y., Zhou Y.H., Xiao Y., Liu X.X., Li X.F., Chen J. (2023). Observation of the hydrogen-dislocation interactions in a high-manganese steel after hydrogen adsorption and desorption. Acta Metall. Sin. Engl. Lett..

[36] Mohapatra S., Mandal A., Das S., Das K. (2024). Study on local strain distribution and grain boundary characteristics of tensile-deformed TRIP-assisted medium manganese steel. Mater. Sci. Eng. A.

[37] Mikami Y., Oda K., Kamaya M., Mochizuki M. (2015). Effect of reference point selection on microscopic stress measurement using EBSD. Mater. Sci. Eng. A.

[38] Ku T.W. (2023). Numerical and experimental investigations on residual stress and hardness within a cold forward extruded preform. Materials.

[39] Zhang J., Hu Z.F., Zhang Z. (2023). EBSD parameter assessment and constitutive models of crept HR3C austenitic steel. J. Iron Steel Res. Int..

[40] Paul V., Ameyama K., Ota-Kawabata M., Ohmura T. (2023). Evaluation of deformation and fracture behavior in 304L austenitic steel harmonic structures through nanoindentation. Steel Res. Int..

[41] Sherar B.W.A., Caldwell E., Ellis P.F., Kane R.D. (2022). Development of the NACE “MR-01-75” and NACE “TM-01-77” standards, Part II: Accelerated material qualification testing in sour environments at near atmospheric pressure. Corrosion.

[42] Dai T., Lippold J.C. (2018). The effect of postweld heat treatment on hydrogen-assisted cracking of F22/625 overlays. Weld. J..

[43] Omweg G.M., Frankel G.S., Bruce W.A., Ramirez J.E., Koch G. (2003). Performance of welded high-strength low-alloy steels in sour environments. Corrosion.

